# Testicular dysfunction at diagnosis in children and teenagers with haematopoietic malignancies improves after initial chemotherapy

**DOI:** 10.3389/fendo.2023.1135467

**Published:** 2023-05-16

**Authors:** Jimena Lopez Dacal, Silvina Prada, Lourdes Correa Brito, Maria Gabriela Ropelato, Maria Gabriela Ballerini, Maria Eugenia Rodriguez, Marcela E. Gutiérrez, Marcela Soria, Lorena Morán, Cristina Ferraro, Patricia Bedecarrás, Guillermo Drelichman, Luis Aversa, Ignacio Bergadá, Rodolfo A. Rey, Romina P. Grinspon

**Affiliations:** ^1^ Centro de Investigaciones Endocrinológicas “Dr. César Bergadá” (CEDIE), CONICET – FEI – División de Endocrinología, Hospital de Niños Ricardo Gutiérrez, Buenos Aires, Argentina; ^2^ Unidad de Hematología, Hospital de Niños Ricardo Gutiérrez, Buenos Aires, Argentina

**Keywords:** AMH, inhibin B, chemotherapy, gonadotropins, hypogonadism, leukemia, lymphoma, testosterone

## Abstract

**Introduction:**

Hematopoietic malignancies are the most frequent type of cancer in childhood. Recent advances in cancer treatment have significantly improved survival until adulthood. There is an extensive literature on the effects of cancer treatment on the gonadal axis in adult survivors of childhood cancer mainly focused on sperm production, but scarce information exists on the immediate impact of cancer and its treatment in boys.

**Objectives:**

In this work, we determined the status of the hypothalamic-pituitary-testicular (HPT) axis function at diagnosis and the immediate impact of chemotherapy at the start of treatment in children and adolescents with hematopoietic malignancies.

**Subjects and methods:**

In a prospective study of 94 boys and adolescents with acute lymphoblastic leukemia (ALL), acute myeloid leukemia (AML) or non-Hodgkin lymphoma (NHL), we determined serum AMH, inhibin B and FSH to assess the gonadotrophin-Sertoli cell component of the HPT axis, and testosterone and LH to evaluate the gonadotrophin-Leydig cell component, at diagnosis and after 3 months of chemotherapy. Secondarily, the general health state was evaluated.

**Results:**

In prepubertal boys, at diagnosis, AMH, inhibin B and FSH were lower compared to the reference population, reflecting an FSH-Sertoli cell axis dysfunction. After 3 months of chemotherapy, all hormone concentrations increased. At pubertal age, at diagnosis, AMH and inhibin B were lower compared to the reference population for Tanner stage, with inappropriately normal FSH, suggesting a primary Sertoli cell dysfunction with insufficient gonadotrophin compensation. The LH-Leydig cell axis was mildly disrupted. After 3 months of chemotherapy, inhibin B and AMH were unchanged while median FSH levels rose to values that exceeded the reference range, indicating a significant impairment of Sertoli cell function. Testosterone normalized concomitantly with an abnormal LH elevation reflecting a compensated Leydig cell impairment. General health biomarkers were impaired at diagnosis and improved after 3 months.

**Conclusion:**

The HPT axis function is impaired in boys with hematopoietic malignancies before the initiation of chemotherapy. There is a primary testicular dysfunction and a concomitant functional central hypogonadism that could be due to an impaired overall health. The HPT axis function improves during the initial 3 months of chemotherapy concomitantly with the general health state. However, in pubertal boys the dysfunction persists as shown by elevated gonadotropin levels after 3 months.

## Introduction

Haematopoietic malignancies represent the commonest type of cancer in childhood (1): approximately 30 cases are diagnosed per million children and adolescents worldwide every year (2). In children younger than 14 years, leukaemia’s account for one third of all cancer types, with acute lymphoblastic leukaemia (ALL) as the most prevalent (3). In adolescents, lymphomas are the most frequent tumour type, representing about 23% of all cancers (3). Presently, more than 80% of haematopoietic malignancies in children and adolescents are timely diagnosed and receive effective treatment. The advances in cancer management have resulted in a significant increase in survival until adulthood, which has raised concern on fertility issues due to gonadal dysfunction (4).

While the assessment of the hypothalamic-pituitary-testicular (HPT) function is usually based upon the study of pituitary gonadotropins, testosterone secreted by Leydig cells and spermatogenesis in adults (5), in childhood mature Leydig cells are absent owing to the lack of LH stimulation and there is no sperm production (6, 7). Before pubertal onset, Sertoli cells are the most active component and anti-Müllerian hormone (AMH) and inhibin B are the preferred biomarkers to assess testicular function (5, 8–10). Serum AMH is high during infancy and childhood and declines at puberty (9, 11, 12) due to a downregulation induced by the increasing concentration of intratesticular testosterone (13). Inhibin B is high during the first 2-3 years of life, decreases though remaining clearly detectable during childhood and increases again during puberty, reflecting both Sertoli cell activity and the development of normal spermatogenesis (8, 9, 14). Both AMH and inhibin B levels are upregulated by FSH (15, 16), and inhibin B is the main responsible for the negative feedback on pituitary FSH production (17).

Most of the evidence of the effects of cancer treatment on the HPT axis relies on studies in adult survivors of childhood cancer and focuses mainly on sperm production (18); scarce information exists on the immediate impact of cancer and its treatment in boys (7, 19, 20). Research has recently addressed the importance of preventing gonadal damage and fertility preservation (21, 22). Sperm cryopreservation is the procedure of choice in adults, but this option is obviously non-existent for prepubertal boys. For these patients, testicular tissue freezing is considered a potential alternative although it is still at an experimental stage (7, 22). In any case, supporting Sertoli cells and Leydig cell precursors would need to be unscathed at the moment of tissue preservation to ensure the success of future auto-transplantation leading to sperm production and androgen secretion, essential during puberty for a normal growth spurt and bone mass accrual (4).

In this work, we aimed to determine the status of the HPT function at diagnosis in children and adolescents with haematopoietic malignancies and the immediate impact of chemotherapy at the start of treatment. In a prospective study of a large cohort of boys and adolescents, we longitudinally assessed serum levels of gonadotrophins and testicular hormones at diagnosis and after the induction phase of chemotherapy.

## Subjects and methods

### Study design and setting

We performed a prospective, analytical, study of a cohort of boys and adolescents diagnosed with haematopoietic malignancies at Ricardo Gutiérrez Children’s Hospitals, a tertiary paediatric public hospital in Buenos Aires, Argentina. Patients were recruited at diagnosis for long-term follow-up in the Unit of Haematology and referred to the Divisions of Endocrinology of Ricardo Gutiérrez, between March 2013 and February 2019. In this interim analysis, the status of the HPT axis was assessed at diagnosis and approximately after the first 3 months of chemotherapy, which corresponds to post-induction phase in the treatment of patients with ALL, acute myeloid leukemia (AML) and most non-Hodgkin lymphoma (NHL).

### Patients

#### Inclusion criteria

Males aged 1-18 years with the diagnoses of ALL, AML or NHL were eligible. The diagnoses were made according to the 3^rd^ and 4^th^ editions of the World Health Organization Classification of Tumors of Hematopoietic and Lymphoid Tissues (23).

#### Exclusion criteria

Patients were excluded if the malignancy overtly involved the central nervous system or the testes, or in any other condition known to affect the hypothalamic-pituitary-testicular axis (12, 24), in order to avoid potential confusion due to known causes of central or primary hypogonadism. Patients for whom serum samples were not available at diagnosis and after 3 months of treatment were excluded.

#### Exposure and follow-up

For patients with ALL, risk stratification and treatment were assigned according to the Acute Lymphoblastic Leukaemia Intercontinental Berlin-Frankfurt-Münster 2009 (ALL IC-BFM 2009 intercontinental trial) (25). Briefly, three risk groups were defined: standard (SR), intermediate (IR) and high risk (HR), as described in **Supplementary Figure 1**. Protocol 8-AML-07 GATLA https://www.gatla.com.ar/images/Protocolos/LMAP_18/LMA_07/1-LMAP07.pdf was followed for patients with AML and protocol Linfoma no Hodgkin Pediátrico GATLA 2017 https://www.gatla.com.ar/images/Protocolos/LNH_2017/GUIA_DE_TRATAMIENTO_LNH_2017.pdf for patients with NHL. The cumulative doses of chemotherapy agents in the first 3 months of treatment are described in **Supplementary Table 1**.

### Outcome measures and definitions

At diagnosis and after 3 months of chemotherapy, the patients were evaluated by a pediatric endocrinologist and were subjected to hormonal assessments. Blood samples were obtained during hospitalization between 07.00 and 10.00 AM, immediately after diagnosis. Both at diagnosis and after 3 months, most patients received oral feeding, and some received iv fluids to maintain a euvolemic state. Patients were grouped according to pubertal stages as defined by Marshall and Tanner (26). Testicular volume was measured by comparison with Prader’s orchidometer.

The main outcome measures of the study were the serum concentrations of AMH, inhibin B and FSH to assess the gonadotrophin-Sertoli cell component of the HPT axis. Secondarily we evaluated serum testosterone and LH, to assess the gonadotrophin-Leydig cell component of the HPT axis, in pubertal patients. Serum hormone levels were expressed as absolute values and as standard deviation scores (SDS) based on age- and Tanner genital stage-matched reference ranges, from a sample of 421 apparently normal males using similar hormone assays, previously published by our group (12, 27) The general health status was assessed by determining hemoglobin, C-reactive protein (CRP), and albumin serum values (28, 29). Additionally, the body mass index (BMI) was calculated as weight (kg)/height (m)^2^, and expressed as standard deviation score (SDS) in boys > 2 years old. (https://zscore.research.chop.edu/calcbmi.php).

### Hormone assays

All hormone assays were performed using fresh samples, except for inhibin B assay that was performed on frozen samples.

#### AMH

Serum AMH was determined using an enzyme-linked immunoassay specific for human AMH (EIA AMH/MIS^®^, Beckman-Coulter Co., Marseilles, France), as previously validated (12, 27). Intra- and inter-assay coefficients of variation were, respectively, 10.5% and 9.4% for a serum AMH concentration of 700 pmol/L (98 ng/mL), and 11.1% and 12.8% for a serum AMH concentration of 7 pmol/L (0.98 ng/mL). When serum AMH levels were undetectable, a value of 1 pmol/L (0.14 ng/mL), corresponding to the limit of quantification (functional sensitivity), was attributed.

#### Inhibin B

Serum inhibin B was determined using an enzyme-linked immunoassay specific for human inhibin B (Inhibin B Gen II ELISA^®^, Immunotech Beckman-Coulter Co., Prague, Czech Republic). Intraassay coefficients of variation were 14% for a serum inhibin B concentration of 111 pg/mL, and 9.8% for 479 pg/mL. Inter-assay coefficients of variation were 14% for a serum inhibin B concentration of 12 pg/mL, and 7.7% for 210 pg/mL. When serum inhibin B levels was undetectable, a value of 7.2 pg/mL, corresponding to the limit of quantification (functional sensitivity), was attributed.

#### Testosterone

Testosterone was determined in serum using an electro-chemiluminescent immunoassay (ECLIA, Roche Diagnostics GmbH, Mannheim, Germany) as described (12). Intra- and inter-assay coefficients of variation were 2.4% and 2.6%, respectively, for a mean testosterone concentration of 176 ng/dL (6.10 nmol/L) and 1.2% and 2.3% for a mean testosterone concentration of 455 ng/dL (15.78 nmol/L). When serum testosterone levels were undetectable, a value of 10 ng/dL (0.347 nmol/L), corresponding to the limit of quantification (functional sensitivity), was attributed.

#### Gonadotropins

LH and FSH were determined using electro-chemiluminescent immunoassays (ECLIA, Roche Diagnostics GmbH, Mannheim, Germany) as described (27). The limits of quantification of both LH and FSH assays were 0.10 IU/L, according to the 2^nd^ National Institute for Biological Standards and Control International Standard (NIBSC IS) 80/552 for LH and the 2^nd^ World Health Organization International Reference Preparation (WHO IRP) 78/549 for FSH. Intra- and inter-assay coefficients of variation were 1.1% and 1.8%, respectively, for a mean LH concentration of 2.8 IU/L and 1.4% and 1.5% for a mean LH concentration of 16.9 IU/L. Intra- and inter-assay coefficients of variation were 1.0% and 4.2%, respectively, for a mean FSH concentration of 14.8 IU/L and 1.1% and 4.1% for a mean FSH concentration of 23.4 IU/L. When serum LH or FSH levels were undetectable, the value of the limit of quantification (functional sensitivity) was attributed.

#### Clinical chemistry

Serum albumin and C-reactive protein (CRP) were measured using immunoturbidimetric assays in Cobas c501 (Roche), and hemoglobin was determined by a colorimetric method in Mindray BC-6800 Plus.

### Statistical analyses

Data distribution was assessed for normality using the Shapiro-Wilks test. Results are expressed as median and interquartile range. For comparisons of median SDS in patients at diagnosis with the theoretical median of 0 SDS in the reference population, we used the parametric one sample t test in case of normal distribution and, in the opposite case, the nonparametric Wilcoxon signed rank test was used. In the same way, depending on the distribution, paired t test or Wilcoxon matched-pairs signed rank test were used to compare between basal hormones and after 3 months of treatment in the individual follow-up of patients. The level of significance was calculated for groups ≥7 patients and set at *P <*0.05. All statistical analyses were performed using GraphPad Prism version 9.4.1 for Windows (GraphPad Software, San Diego, CA, USA).

The calculation of the study sample size was performed to determine the proportion of patients with Sertoli cell dysfunction up to 36 months of follow-up in the longitudinal cohort of boys and adolescents diagnosed with hematopoietic malignancies. All of the patients of the longitudinal study who had clinical and hormonal assessments at diagnosis and after 3 months of chemotherapy were included in the present study. Since this is an interim study of the whole cohort, a specific sample size calculation was not performed.

### Ethical issues

Research was conducted in accordance with principles of the Declaration of Helsinki and local regulations on observational studies with human subjects. The study protocol was approved by the Institutional Review Board of the Ricardo Gutiérrez Children’s Hospital, Buenos Aires (# CEI 14.07). All patients or their parents, as appropriate, provided informed consent.

## Results

### Characteristics of the study sample

**Figure 1:**
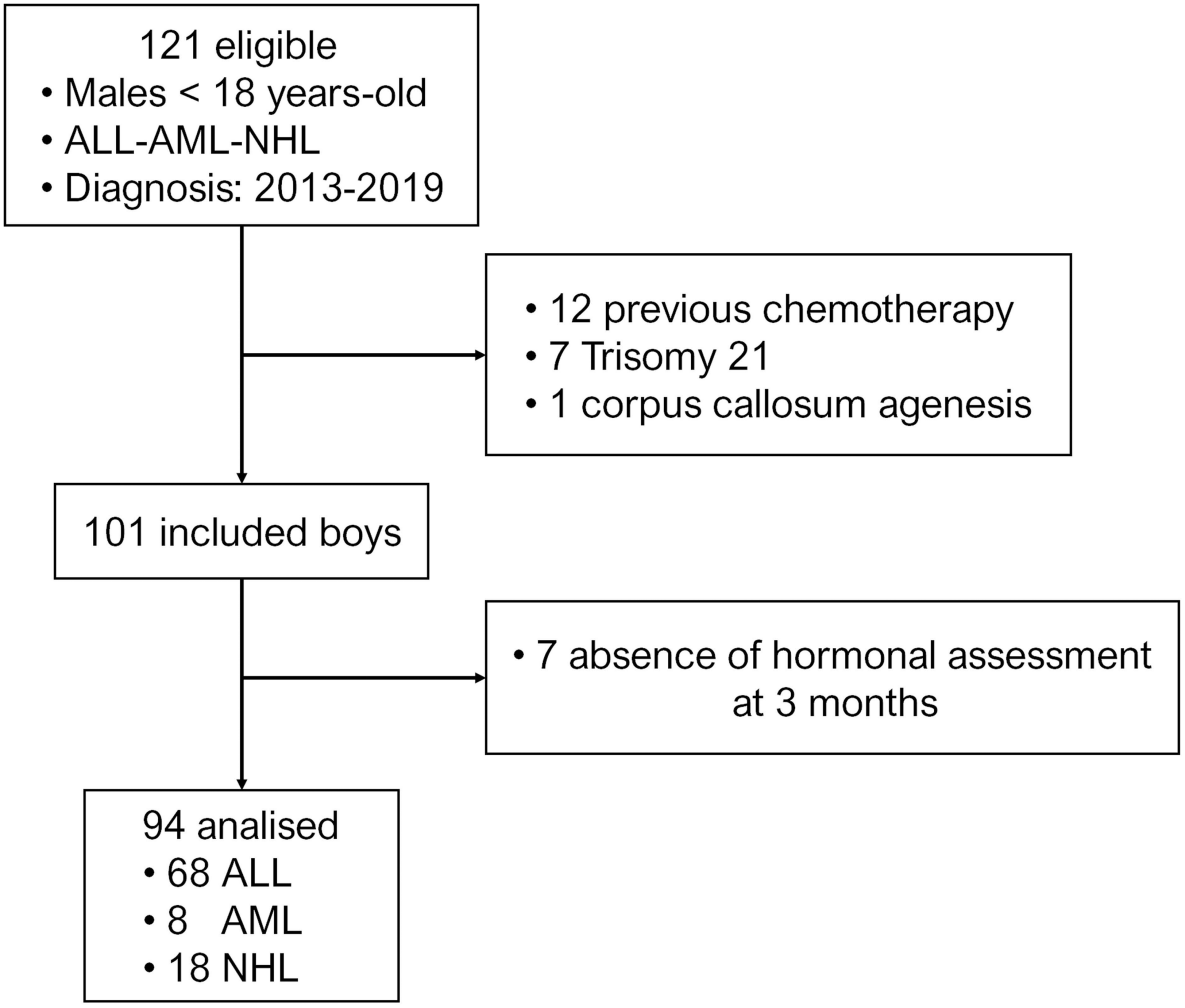
Flow-chart of patients included in the study.

Twenty out of 121 eligible patients were not included for the following reasons: 12 initiated chemotherapy treatment prior to the first hormonal evaluation, 7 had trisomy 21 and 1 had corpus callosum agenesis (**Figure 1**). From the 101 patients with hematopoietic malignancies included in the long-term follow-up cohort, a serum sample at 3 months was not available in 7 patients, who were therefore excluded. From the 94 patients analyzed in the present study, 70 (74.5%) were prepubertal. As expected, ALL represented the most frequent diagnosis, and boys with ALL were younger than those with AML or NHL; the analysis of the results of the whole study were made according to the pubertal status (**Table 1**). The median time between diagnosis and post-chemotherapy serum sample was 2.9 months (interquartile range: 2.6-3.3).

**Table 1:** Characteristics of the study sample.

Pubertal status	Diagnosis	Risk	n	%	Age (years)
Whole cohort			94		6.9 (0.5-17.6)
	ALL AML NHL	SR/IR HR	44 24 8 18	47% 26% 9% 19%	4.5 (1.5-16.8) 8.9 (0.5-16.4) 11.9 (1.8-14.5) 8.8 (1.9-17.6)
Prepubertal			70		4.4 (0.5-14.3)
	ALL AML NHL	SR/IR HR	37 18 3 12	53% 26% 4% 17%	4.4 (1.5-14.3) 5.2 (0.5-10.9) 5.9 (1.7-7.8) 6.4 (1.9-10.1)
Pubertal			24		13.7 (9.3-17.6)
	ALL AML NHL	SR/IR HR	7 6 5 6	29% 25% 21% 25%	13.5 (11.8-16.8) 14.9 (11.7-16.4) 13 (11.9-14.5) 15.4 (9.3-17.6)

ALL, acute lymphoblastic leukemia; AML, acute myeloid leukemia; NHL, non-Hodgkin lymphoma; SR, standard risk; IR, intermediate risk; HR, high risk.

### Prepubertal boys

**Figure 2:**
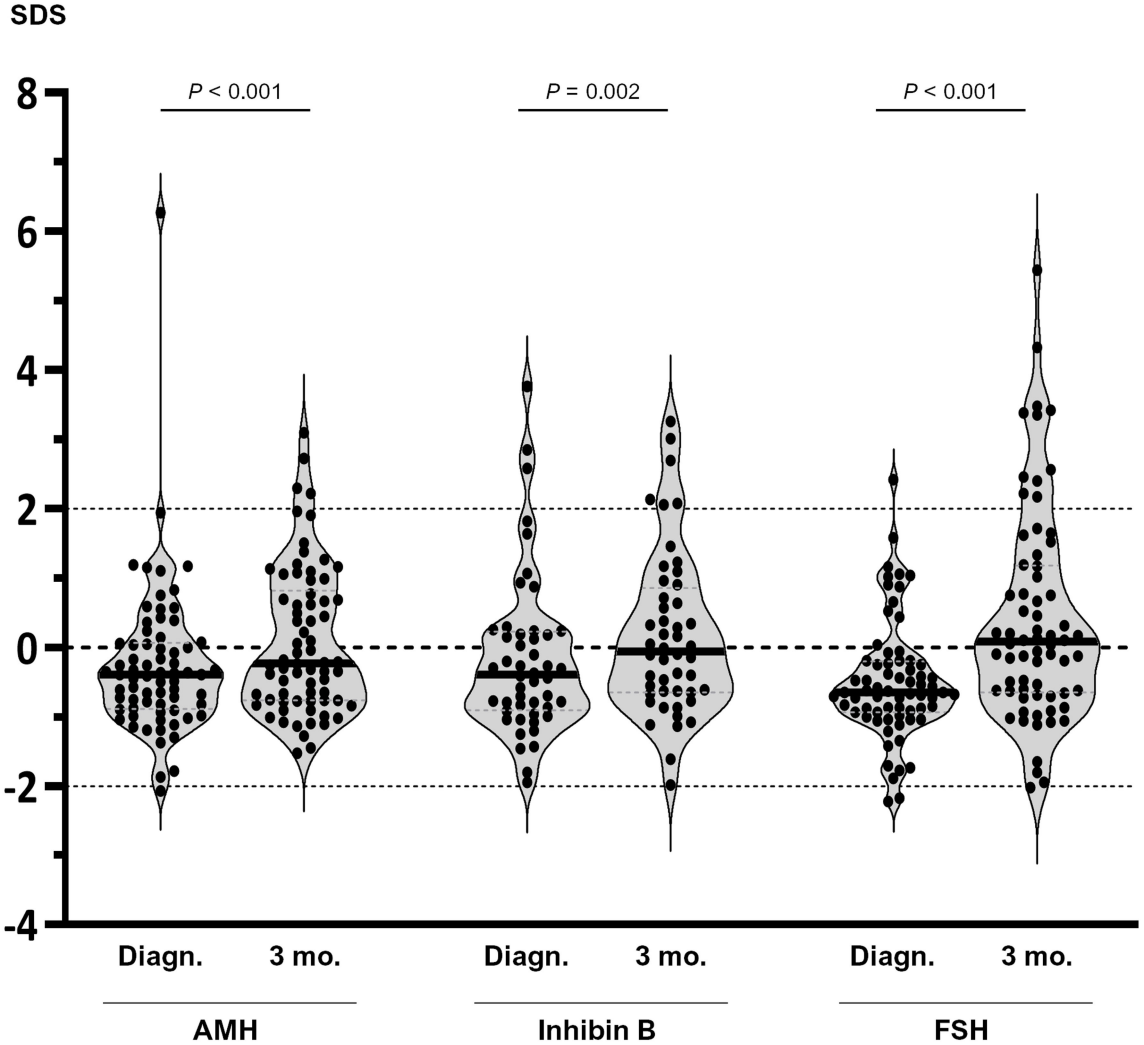
Serum levels of AMH, inhibin B and FSH in prepubertal boys with hematopoietic malignancies at diagnosis (Diagn.) and after 3 months (3 mo.) of chemotherapy. Values are expressed as standard deviation scores (SDS). Within each violin, the line represents the median. Comparisons between diagnosis and 3 months were analysed using the Wilcoxon matched-pairs signed rank test.

Serum levels of AMH, inhibin B and FSH were measured to assess the functional status of the gonadotrophin-Sertoli cell component of the HPT axis. Given that reference hormone levels change during childhood and puberty, we analyzed our results using SDS. Nonetheless, absolute hormone levels are provided in **Supplementary Table 2**. At diagnosis, serum levels of all 3 hormones were significantly below the median levels expected for the reference population, as analyzed using the Wilcoxon signed rank test: AMH (median -0.39 SDS, IQR -0.89 to 0.06, P<0.001), inhibin B (-0.39 SDS; IQR -0.91 to 0.23, P=0.035) and FSH (-0.65 SDS, IQR -0.94 to 0.20, P<0.001) (**Figure 2**). Serum levels below 0 SDS were observed in 70% of the boys for AMH, 64.6% for inhibin B and 83.8% for FSH. However, values below -2 SDS were found in only 1 patient for AMH and 2 boys for FSH. Altogether, these results suggested the existence of a mild impairment of the FSH-Sertoli cell axis function at diagnosis in prepubertal boys with hematopoietic malignancies. After 3 months of chemotherapy, there was an increase in median serum levels of all 3 hormones: AMH (-0.23 SDS, IQR -0.76 to 0.82), inhibin B (-0.06 SDS, IQR -0.94 to 0.20) and FSH (0.08 SDS, IQR -0.65 to 1.19). FSH levels were above 2 SDS in 11 patients (16.2%) at 3 months of treatment.

**Figure 3:**
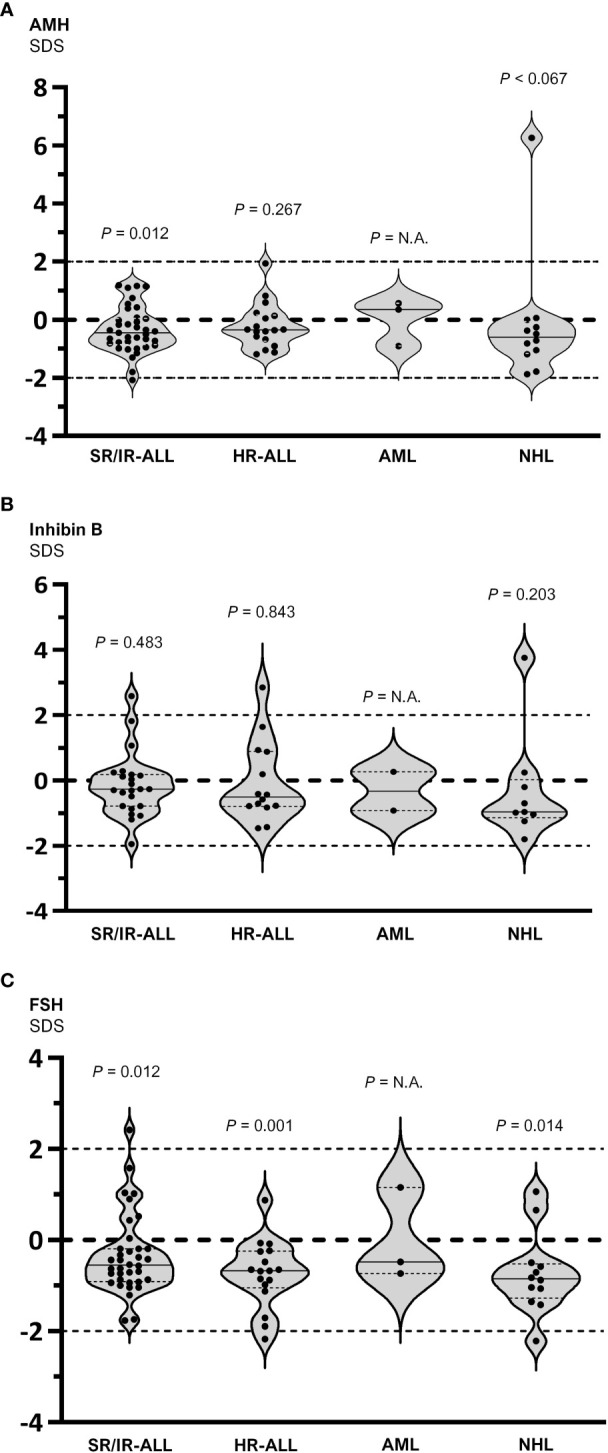
Serum hormone levels in prepubertal boys with hematopoietic malignancies grouped by diagnosis: standard/intermediate risk (SR/IR) and high risk (HR) acute lymphoblastic leukemia (ALL), acute myeloid leukemia (AML) and non-Hodgkin lymphoma (NHL). Values are expressed as standard deviation scores (SDS) and compared to the theoretical value of 0 SDS. In the group with AML, a statistical analysis was not applicable (N.A.) given the insufficient number of observations. Within each violin, the full line represents the median and the dotted lines the 25^th^ and 75^th^ centiles. **(A)** AMH: one sample t test was used for SR/IR and HR ALL, and Wilcoxon signed rank test for NHL. **(B)** Inhibin B: one sample t test was used for SR/IR and HR ALL, and Wilcoxon signed rank test for NHL. **(C)** FSH: a Wilcoxon signed rank test was used for SR/IR ALL, and a one sample t test for HR ALL and NHL.

**Figure 4:**
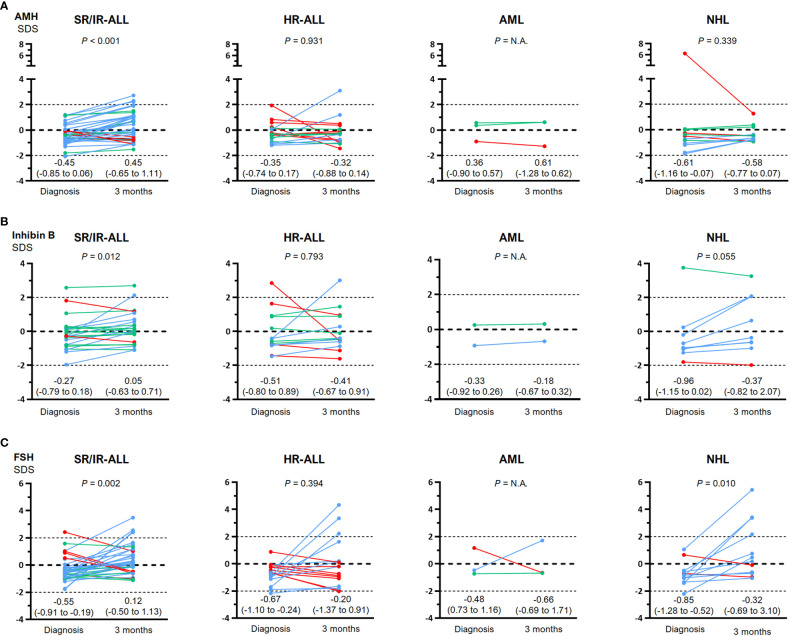
Comparison between serum hormone levels at diagnosis and after 3 months of chemotherapy in individual prepubertal boys with hematopoietic malignancies: standard/intermediate risk (SR/IR) and high risk (HR) acute lymphoblastic leukemia (ALL), acute myeloid leukemia (AML) and non-Hodgkin lymphoma (NHL). Values are expressed as standard deviation scores (SDS). Light blue lines indicate an increase, green lines denote no change and red lines, a decrease in hormone levels. **(A)**: AMH. For each individual, serum AMH was considered to increase or decrease when the difference between diagnosis and 3 months was >20% or <20%, respectively. Statistical analyses were performed using a paired t test for SR/IR and HR ALL, and Wilcoxon matched-pairs signed rank test for NHL. **(B)**: Inhibin B For each individual, serum inhibin B was considered to increase or decrease when the difference between diagnosis and 3 months was >25% or <25%, respectively. Statistical analyses were performed using paired t test for SR/IR and HR ALL, and Wilcoxon matched-pairs signed rank test for NHL. **(C)**: FSH. For each individual, serum FSH was considered to increase or decrease when the difference between diagnosis and 3 months was >5% or <5%, respectively. Statistical analyses were performed using Wilcoxon matched-pairs signed rank for SR/IR and HR ALL, and a paired t test for NHL. In all the boys with AML, statistical analyses were not applicable (N.A.) given the insufficient number of observations.

When analyzed by diagnosis, baseline AMH was decreased in patients with SR/IR-ALL (-0.45 SDS; IQR -0.85 to 0.06) but not in those with HR-ALL (-0.35 SDS; IQR -0.74 to 0.17) or NHL (-0.61 SDS; IQR -1.16 to -0.07) (**Figure 3A**). An increase to 0.45 SDS (-0.65 to 1.11 SDS) was observed in serum AMH after 3 months of chemotherapy in patients with SR/IR ALL, but no significant changes were seen in boys with HR-ALL or NHL (**Figure 4A**). Inhibin B was -0.27 SDS (-0.79 to 0.18) in SR/IR ALL, -0.51 SDS (-0.80 to 0.89) in HR-ALL and -0.96 SDS (-1.15 to 0.02) in NHL at diagnosis (**Figure 3B**). After 3 months of chemotherapy, inhibin B increased in patients with SR/IR ALL patients (0.05 SDS; IQR -0.63 to 0.71 SDS), but did not significantly change in boys with HR-ALL or NHL (**Figure 4B**). Regarding FSH, significantly decreased levels were observed in all groups: SR/IR-ALL (-0.55, IQR -0.91 to -0.19), HR-ALL (-0.67, IQR -1.10 to -0.24) and NHL (-0.85; IQR -1.28 to -0.52) (**Figure 3C**). After 3 months of chemotherapy, FSH increased to 0.12 SDS (-0.50 to 1.13) in patients with SR/IR-ALL and to 0.32 SDS (-0.69 to 3.10) in boys with NHL (**Figure 4C**).

Although our initial aim was to determine the immediate impact of chemotherapy in the first 3 months, we sought to determine whether the changes in hormone levels remained stable. Data shown in **Supplementary Table 4** indicate that outcomes were overall stable until at least the 6^th^ month of treatment.

### Pubertal boys

To assess the functional status of the pituitary-Sertoli cell axis, we used serum levels of FSH, inhibin B and AMH, whereas for the pituitary-Leydig cell axis we analyzed serum levels of LH and testosterone. Since reference hormone levels change according to Tanner stage, SDS were used for the analysis. Nonetheless, absolute hormone levels are provided in **Supplementary Table 3**.

**Figure 5:**
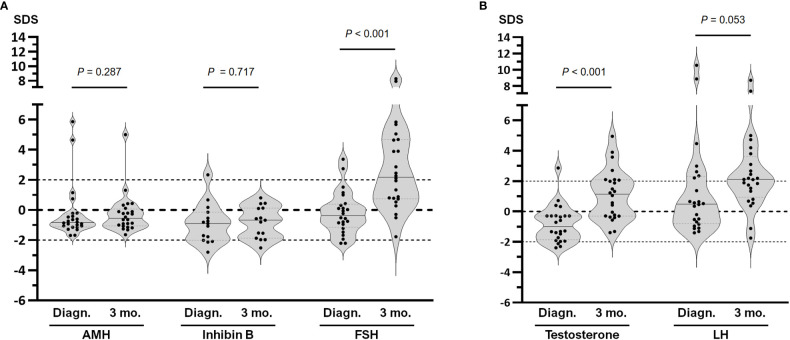
Hormone serum levels in pubertal boys with hematopoietic malignancies at diagnosis (Diagn.) and after 3 months of chemotherapy (3 mo.). **(A)** Pituitary-Sertoli cell axis, including AMH, inhibin B and FSH. **(B)** Pituitary-Leydig cell axis, including testosterone and LH. Values are expressed as standard deviation scores (SDS); comparison between Diagn. and 3 mo. were analysed using the Wilcoxon signed rank test for AMH, testosterone and LH, and paired t test for inhibin B and FSH. Within each violin, the line represents the median.

At diagnosis, values significantly below the median levels expected for the reference population were seen for serum inhibin B (-0.90 SDS; IQR -2.01 to -0.14, P=0.019, one sample t test) and AMH (median -0.83 SDS, IQR -1.12 to -0.26, P=0.029, Wilcoxon signed rank test), whereas median FSH was within the reference range (-0.37 SDS, IQR -1.14 to 0.39, P=0.519, one sample t test). Serum inhibin was below 0 SDS in 80.0% of the boys and 4 patients (26.7%) had levels below -2 SDS, while AMH was below 0 SDS in 83.3% but none was below -2 SDS. After 3 months of chemotherapy, median inhibin B (-0.67 SDS; -1.87 to 0.13, P=0.010, one sample t test) and AMH (-0.55 SDS; -1.05 to 0.07, P=0.028, Wilcoxon signed rank test) remained below 0 SDS, with no significant changes compared to the basal state, whereas median FSH (2.19 SDS; 0.74 to 4.68, P<0.001, one sample t test) increased significantly to abnormally high levels. FSH was above +2 SDS in 13 patients (54.2%) (**Figure 5A**). Altogether, these results suggest that the Sertoli cell component is disrupted at diagnosis and the dysfunction persists after 3 months of chemotherapy, inducing an hypergonadotropic state.

The pituitary-Leydig cell axis showed decreased levels of testosterone (-1.00 SDS; -1.86 to -0.29, P=0.004, Wilcoxon signed rank test) with normal LH (0.49 SDS; -0.80 to 2.32, P=0.179, Wilcoxon signed rank test) at diagnosis. Serum testosterone was below 0 SDS in 83.3% of the patients and 3 patients (12.5%) showed serum testosterone below <2 SDS. After 3 months of chemotherapy, median testosterone increased significantly (1.15 SDS; -0.30 to 2.09, P=0.003, one sample t test), while LH showed median levels above 0 SDS (2.12 SDS; 0.94 to 3.63, P<0.001, one sample t test); LH was above +2 SDS in 13 patients (54.2%) (**Figure 5B**). These observations suggest that Leydig cell function was impaired at diagnosis and improved after 3 months of chemotherapy in association with high levels of LH.

Individual assessments by diagnosis are shown in **Supplementary Figures 2**, **3**. Statistical analyses were not possible due to the low number of observations in each subgroup. Data in **Supplementary Table 5** indicate that changes were overall stable until 6 months of chemotherapy.

### General health status

In prepubertal boys, median serum hemoglobin and albumin were low at diagnosis and increased after 3 months of chemotherapy, whereas median C-reactive protein was high and normalized after 3 months (**Supplementary Table 6**). In pubertal patients too, hemoglobin and albumin were low and C-reactive protein was high at diagnosis. While hemoglobin did not significantly change at 3 months, albumin increased, and C-reactive protein decreased to normal levels. Altogether, these results suggest that the general health status was compromised at diagnosis and improved after 3 months of chemotherapy. The prevalence of boys in whom the hormonal status improved at 3 months was similar in those who showed a better health status, as reflected in C-reactive protein levels, and in those who did not (**Supplementary Table 7**).

## Discussion

The results of the present study show that, at diagnosis, a large proportion of prepubertal boys with ALL, AML or NHL had lower AMH, inhibin B and FSH concentrations compared to the reference population, reflecting an FSH-Sertoli cell axis dysfunction before any treatment was initiated. After 3 months of chemotherapy, all hormone concentrations increased, especially in the prepubertal boys with SR/IR-ALL. At pubertal age, boys with hematopoietic malignancies had lower AMH and inhibin B concentrations compared to the reference population for Tanner stage, with inappropriately normal FSH levels at diagnosis, reflecting a primary Sertoli cell dysfunction with inadequate gonadotrophin compensation. After 3 months of chemotherapy, inhibin B and AMH did not recover and FSH increased to high levels, suggesting a significant impairment of the Sertoli cell function. The LH-Leydig cell axis function was slightly impaired at diagnosis. After 3 months of chemotherapy, the increase in testosterone concomitantly with an elevation in LH to supraphysiological levels probably reflects a compensated Leydig cell impairment.

AMH and inhibin B are produced by Sertoli cells, the most active cell population during childhood. The low concentrations of these two peptides at diagnosis in prepubertal boys with leukemias or non-Hodgkin lymphoma could be due to primary Sertoli cell dysfunction. Our results in boys with hematopoietic malignancies are in line with a previous report in a large series of prepubertal males with various cancer types (30). Testicular dysfunction may be due to gonadal infiltration by tumor cells and/or by the action of inflammatory cytokines on gonadal tissue. Indeed, testicular infiltration has been extensively described in boys with leukemia (31–33), and CXCL12 expressed in Sertoli cells has been identified as part of a paracrine signaling system with the potential to sustain the migration and persistence of leukemic cells in the testis (34). The coexistence of low FSH levels with Sertoli cells hypofunction could indicate a concomitant central hypogonadism in prepubertal boys, that is, a combined primary and central hypogonadism (24). Central hypogonadism can occur in response to a generalized disease that deteriorates the general condition. In our series of patients, low serum hemoglobin and albumin together with increased C-reactive protein at diagnosis favor the hypothesis of an affected general health status. However, the improvement in the hormone levels in a subset of patients without a clear recovery of the health status suggests the involvement of additional underlying mechanisms, for instance an effect of chemotherapy on gonadal infiltration by leukemic cells.

In boys with congenital central hypogonadism, low inhibin B and AMH levels indicate a Sertoli cell hypoplasia secondary to pituitary gonadotropin deficiency reflecting a long-term insufficient FSH activity on Sertoli cell proliferation and function (15). Conversely, acquired central hypogonadism results in a less significant effect on Sertoli cell biomarkers, likely due to the fact that sufficient FSH activity induced normal Sertoli cell proliferation during fetal and neonatal periods leading to an adequate mass of Sertoli cells, as reflected by normal testicular volume in most cases (35).

The increase in FSH, AMH and inhibin B after 3 months of treatment in the prepubertal boys was unexpected given that chemotherapy is believed to have gonadotoxic effects, especially when alkylating agents are used (36). Our results suggest an improvement of the FSH-Sertoli cell axis during the initial phases of chemotherapy, which coincides with an improvement in the biomarkers of general health status. Noteworthy, testicular function, as reflected by serum AMH and inhibin B, improved in patients with SR/IR ALL but not in those with HR-ALL, AML or NHL. The lower number of patients with HR-ALL, AML and NHL may deter the detection of a statistically significant difference. Alternatively, the more significant increase in FSH levels in patients with SR/IR-ALL could underlie the better response in AMH and inhibin B. This is the first study to report an improvement of the HPT axis function in the initial months of chemotherapy in boys with hematopoietic malignancies.

In boys diagnosed during puberty, the endocrine function of the testis was affected both in the interstitial (Leydig cell) and the seminiferous tubule (Sertoli cell) compartments, in agreement with previous findings in all cancer types (30) and in a smaller study including specifically adolescents with leukemia or lymphoma (37). Testicular function in pubertal boys with Hodgkin disease was described to be more adversely affected by chemotherapy than in prepubertal boys long time ago (38). Although chemotherapy regimens have changed, it is interesting to point out that this pioneering observation focused on germ cell depletion during pubertal maturation. Here, we add the value of Sertoli cell biomarkers. The production of AMH and inhibin B differ in pubertal boys: AMH is produced exclusively by Sertoli cells whereas inhibin B is secreted as a cooperative action of Sertoli and germ cells. The low concentrations of these two peptides at diagnosis could be due to a primary Sertoli cell dysfunction (low AMH) and germ cell damage (low inhibin B). The coexistence of inappropriately normal FSH levels with low inhibin B in pubertal boys could indicate, like in prepubertal boys, a combined hypogonadism. The same analysis applies to the LH-Leydig cell axis, where low testosterone coexisted with inappropriately normal LH. Conversely, after 3 months of chemotherapy, the behavior of the two compartments differed. Despite an increase of both FSH and LH to supraphysiological levels, Inhibin B and AMH did not recover whereas median testosterone exceeded 1 SDS. This suggests a dissociated hypogonadism, with a compensated Leydig cell activity and an insufficient seminiferous tubule function. A more toxic effect of chemotherapy on the germ cell population (39, 40) could explain the insufficient increase in serum inhibin B. Alternatively, Sertoli cells could be primarily affected. In fact, there is an FSH-induced peak of Sertoli cell proliferation in the initial phases of puberty (41), and proliferating cells are more sensitive to most chemotherapy agents. As regards the LH-Leydig cell loop, it could also be influenced by a Sertoli-cell paracrine dysregulation. Indeed, impaired Sertoli cell function, either primary or due to germ cell depletion, may result in increased activin secretion and signaling on neighboring Leydig cells, thus leading to decreased testosterone production requiring higher LH stimulation (42).

In conclusion, the HPT axis is impaired at diagnosis in boys with hematopoietic malignancies. There seems to be a combined hypogonadism, with a primary testicular dysfunction possibly due to gonadal infiltration by tumor cells and/or the effect of inflammatory factors on the gonad, and a concomitant functional central hypogonadism that could be due to an impaired overall health. The HPT axis function improves during the initial 3 months of chemotherapy, with certain nuances according to age and testicular compartment, concomitantly with the general health state.

## Data availability statement

The raw data supporting the conclusions of this article will be made available by the authors, without undue reservation.

## Ethics statement

The studies involving human participants were reviewed and approved by Comité de Ética en Investigación, Hospital de Niños Ricardo Gutiérrez. All patients or their parents, as appropriate, provided informed consent.

## Author contributions

RG, RR, GD, LA, MGR and IB conceived the study design; JD, SP, LB, MB, MER, MEG, MS, LM, CF and PB collected clinical and laboratory data; JD, SP, LB, RR and RG analyzed the data; JD, RR and RG drafted the manuscript; All authors contributed to the article and approved the submitted version.

## Supplementary material

Supplementary Figure 1 Risk group definition based on age, white blood cell count, cytogenetics, response on peripheral blood at day 8, on bone narrow at days 15 and 33 and flow cytometric-minimal residual disease according to ALL IC-BFM 2009 protocol. BM: bone marrow, FCM-MRD: Flow cytometric-minimal residual disease, M1: <5% bone marrow blasts, M2: 5-25% bone marrow blasts, M3: >25% bone marrow blasts, PB: peripheral blood, WBC: white blood cells.

Supplementary Figure 2 Comparison of pituitary-Sertoli cell hormone levels at diagnosis and after 3 months of chemotherapy in individual pubertal boys with hematopoietic malignancies: standard/intermediate risk (SR/IR) and high risk (HR) acute lymphoblastic leukemia (ALL), acute myeloid leukemia (AML) and non-Hodgkin lymphoma (NHL). Values are expressed as standard deviation scores (SDS, median and interquartile range). Light blue lines indicate an increase, green lines denote no change and red lines, a decrease in hormone levels. **(A)** AMH. For each individual, serum AMH was considered to increase or decrease when the difference between diagnosis and 3 months was >20% or <20%, respectively. **(B)** Inhibin B. For each individual, serum inhibin B was considered to increase or decrease when the difference between diagnosis and 3 months was >25% or <25%, respectively. **(C)** FSH. For each individual, serum FSH was considered to increase or decrease when the difference between diagnosis and 3 months was >5% or <5%, respectively. Statistical analyses were not performed given the insufficient number of observations in each subgroup.

Supplementary Figure 3 Comparison of pituitary-Leydig cell hormone levels at diagnosis and after 3 months of chemotherapy in individual pubertal boys with hematopoietic malignancies: standard/intermediate risk (SR/IR) and high risk (HR) acute lymphoblastic leukemia (ALL), acute myeloid leukemia (AML) and non-Hodgkin lymphoma (NHL). Values are expressed as standard deviation scores (SDS, median and interquartile range). Light blue lines indicate an increase, green lines denote no change and red lines, a decrease in hormone levels. **(A)** Testosterone. For each individual, serum testosterone was considered to increase or decrease when the difference between diagnosis and 3 months was >5% or <5%, respectively. **(B)** LH. For each individual, serum LH was considered to increase or decrease when the difference between diagnosis and 3 months was >3% or <3%, respectively. Statistical analyses were not performed given the insufficient number of observations in each subgroup.
