# Supplementary figures and images for: Testicular dysfunction at diagnosis in children and teenagers with haematopoietic malignancies improves after initial chemotherapy
Source: Front Endocrinol (Lausanne). 2023 May 16;14:1135467. doi: 10.3389/fendo.2023.1135467 (PMC10228689; doi:10.3389/fendo.2023.1135467)

Supplementary Figure 1

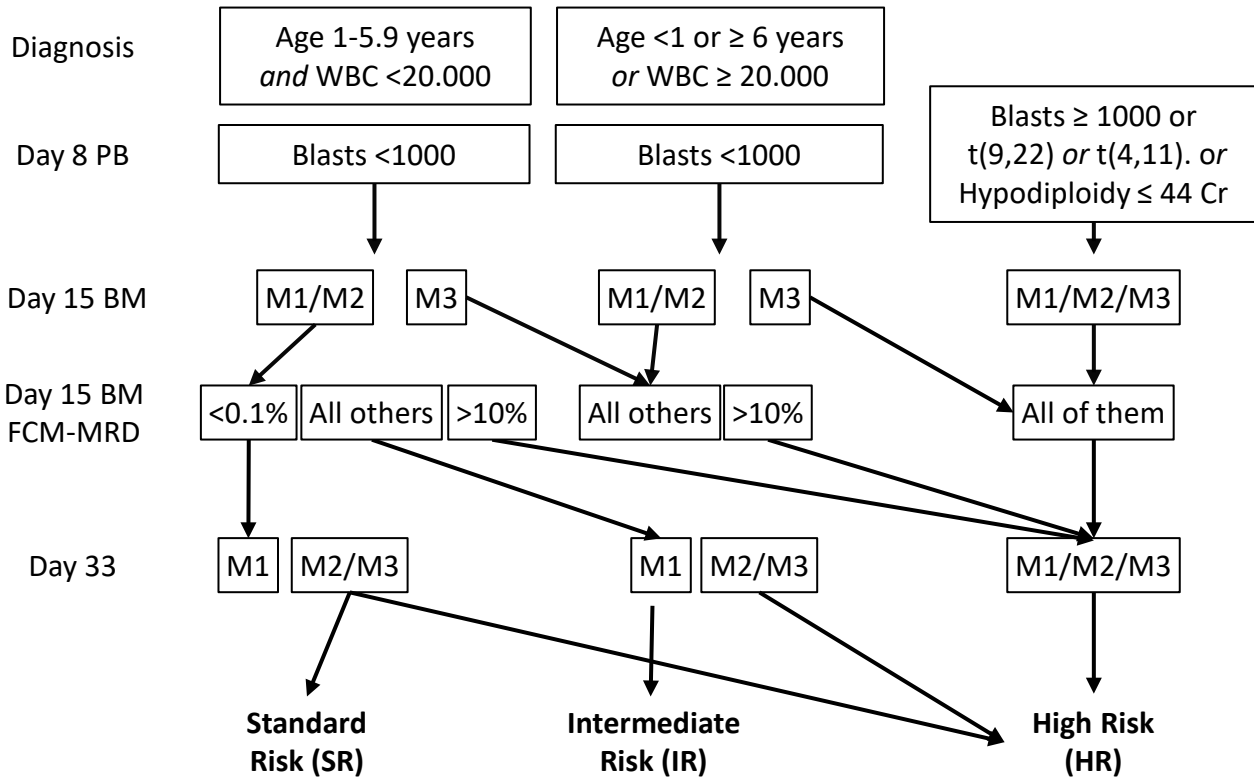

Supplement: Supplementary Figure 1 — Risk group definition based on age, white blood cell count, cytogenetics, response on peripheral blood at day 8, on bone narrow at days 15 and 33 and flow cytometric-minimal residual disease according to ALL IC-BFM 2009 protocol. BM: bone marrow, FCM-MRD: Flow cytometric-minimal residual disease, M1: <5% bone marrow blasts, M2: 5-25% bone marrow blasts, M3: >25% bone marrow blasts, PB: peripheral blood, WBC: white blood cells. [file Image_1.pdf]

# Supplementary Figure 2

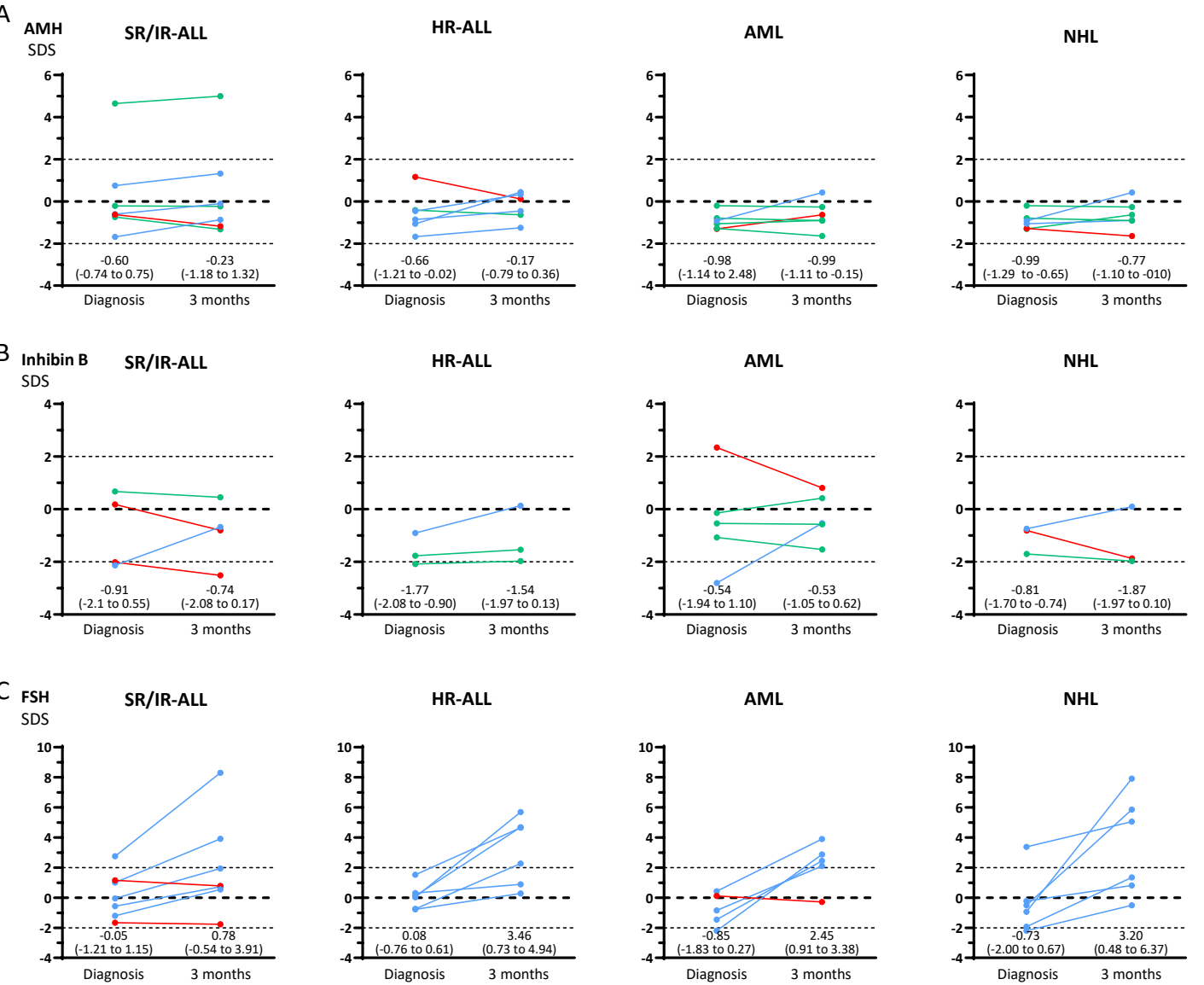

Supplement: Supplementary Figure 2 — Comparison of pituitary-Sertoli cell hormone levels at diagnosis and after 3 months of chemotherapy in individual pubertal boys with hematopoietic malignancies: standard/intermediate risk (SR/IR) and high risk (HR) acute lymphoblastic leukemia (ALL), acute myeloid leukemia (AML) and non-Hodgkin lymphoma (NHL). Values are expressed as standard deviation scores (SDS, median and interquartile range). Light blue lines indicate an increase, green lines denote no change and red lines, a decrease in hormone levels. (A) AMH. For each individual, serum AMH was considered to increase or decrease when the difference between diagnosis and 3 months was >20% or <20%, respectively. (B) Inhibin B. For each individual, serum inhibin B was considered to increase or decrease when the difference between diagnosis and 3 months was >25% or <25%, respectively. (C) FSH. For each individual, serum FSH was considered to increase or decrease when the difference between diagnosis and 3 months was >5% or <5%, respectively. Statistical analyses were not performed given the insufficient number of observations in each subgroup. [file Image_2.pdf]

Supplementary Figure 3

A

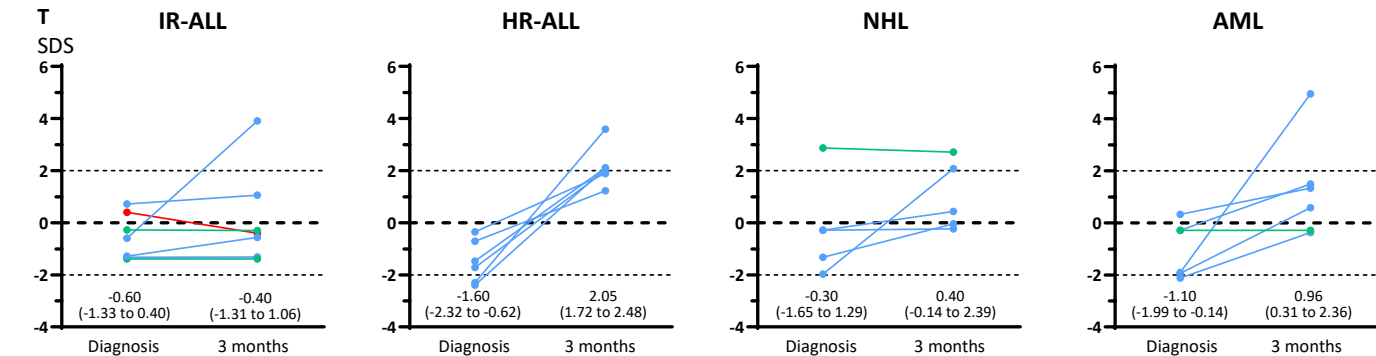

B

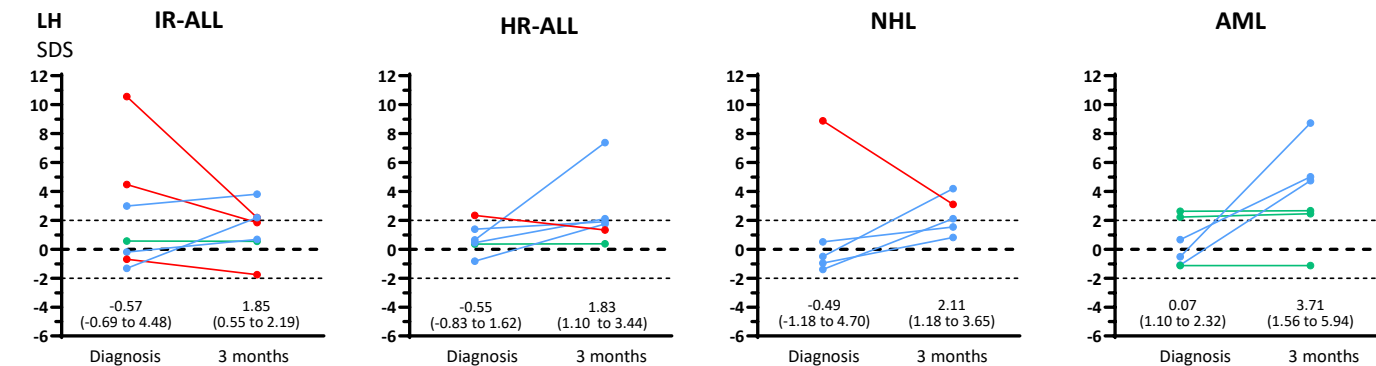

Supplement: Supplementary Figure 3 — Comparison of pituitary-Leydig cell hormone levels at diagnosis and after 3 months of chemotherapy in individual pubertal boys with hematopoietic malignancies: standard/intermediate risk (SR/IR) and high risk (HR) acute lymphoblastic leukemia (ALL), acute myeloid leukemia (AML) and non-Hodgkin lymphoma (NHL). Values are expressed as standard deviation scores (SDS, median and interquartile range). Light blue lines indicate an increase, green lines denote no change and red lines, a decrease in hormone levels. (A) Testosterone. For each individual, serum testosterone was considered to increase or decrease when the difference between diagnosis and 3 months was >5% or <5%, respectively. (B) LH. For each individual, serum LH was considered to increase or decrease when the difference between diagnosis and 3 months was >3% or <3%, respectively. Statistical analyses were not performed given the insufficient number of observations in each subgroup. [file Image_3.pdf]
